# Data-driven inference for the spatial scan statistic

**DOI:** 10.1186/1476-072X-10-47

**Published:** 2011-08-02

**Authors:** Alexandre CL Almeida, Anderson R Duarte, Luiz H Duczmal, Fernando LP Oliveira, Ricardo HC Takahashi

**Affiliations:** 1 Campus Alto Paraopeba, Universidade Federal de São João del Rei, Ouro Branco/MG, Brazil; 2 Department of Mathematics, Universidade Federal de Ouro Preto, Ouro Preto/MG, Brazil; 3 Department of Statistics, Universidade Federal de Minas Gerais, Campus Pampulha, Belo Horizonte/MG, Brazil; 4 Department of Mathematics, Universidade Federal de Minas Gerais, Campus Pampulha, Belo Horizonte/MG, Brazil; 5 Graduate Program in Electrical Engineering, Universidade Federal de Minas Gerais, Campus Pampulha, Belo Horizonte/MG, Brazil

## Abstract

**Background:**

Kulldorff's spatial scan statistic for aggregated area maps searches for clusters of cases without specifying their size (number of areas) or geographic location in advance. Their statistical significance is tested while adjusting for the multiple testing inherent in such a procedure. However, as is shown in this work, this adjustment is not done in an even manner for all possible cluster sizes.

**Results:**

A modification is proposed to the usual inference test of the spatial scan statistic, incorporating additional information about the size of the most likely cluster found. A new interpretation of the results of the spatial scan statistic is done, posing a modified inference question: what is the probability that the null hypothesis is rejected for the original observed cases map with a most likely cluster of size k, taking into account only those most likely clusters of size k found under null hypothesis for comparison? This question is especially important when the p-value computed by the usual inference process is near the alpha significance level, regarding the correctness of the decision based in this inference.

**Conclusions:**

A practical procedure is provided to make more accurate inferences about the most likely cluster found by the spatial scan statistic.

## Background

### Introduction

Spatial cluster analysis is considered an important technique for the elucidation of disease causes and epidemiological surveillance [1]. Kulldorff's spatial scan statistic, defined as a likelihood ratio, is the usual measure of the strength of geographic clusters [2,3]. The circular scan [4], a particular case of the spatial scan statistic, is currently the most used tool for the detection and inference of spatial clusters of disease.

The spatial scan statistic considers a study region *A *divided into *m *areas, with total population *N *and *C *total cases. A zone is any collection of areas. The null hypothesis assumes that there are no clusters and the cases are uniformly distributed, such that the expected number of cases in each area is proportional to its population. A commonly used model assumes that the number of cases in each area is Poisson distributed proportionally to its population. Let *c_z _*be the number of observed cases and *n_z _*be the population of the zone *z*. The expected number of cases under null hypothesis is given by *μ_z _*= *C*(*n_z_*/*N *). The relative risk of *z *is *I*(*z*) = *c_z_*/*μ_z _*and the relative risk outside *z *is *O*(*z*) = (*C *- *c_z_*)/(*C *- *μ_z_*). If *L*(*z*) is the likelihood function under the alternative hypothesis and *L*_0 _is the likelihood function under the null hypothesis, the logarithm of the likelihood ratio for the Poisson model is given by:(1)

*LLR*(*z*) is maximized over the chosen set *Z *of potential zones *z*, identifying the zone that constitutes the *most likely cluster *(MLC). A derivation of this model can be found in [2]. When the set *Z *contain the zones defined by circular windows of different radii and centers, max_*z*∈*Z *_*LLR*(*z*) is the circular scan statistic. Other possible choices for *Z *include the set of elliptic clusters [5], or even the set of irregularly shaped connected clusters [6,7].

The statistical significance of the original MLC of observed cases must be calculated employing Monte Carlo simulations to build an empirical distribution of the obtained max_*z*∈_*_Z _LLR*(*z*) values under null hypothesis [8], because its analytical expression is generally not known. Simulated cases are randomly distributed over the study region such that each area receives, on average, a number of cases proportional to its population. The statistical significance of the original MLC of observed cases is tested comparing its LLR value with LLR values of the corresponding MLCs obtained for each Monte Carlo replication. In each one of those replications, the MLC will be chosen in the set of circular clusters of every possible size centered on every area of the study region, meaning that the LLR(z) value is the only selected feature used to compare the original MLC with the random ones under null hypothesis. The scan statistic is then computed for the MLC. This procedure is repeated *B *times, obtaining the empirical distribution of the max*_z_*_∈_*_Z _LLR*(*z*) values. Let *Y *be the number of times that those values are greater than the *LLR *of the original MLC of observed cases. The p-value of the original MLC is computed as (*Y *+ 1)/*B*. In the following, the empirical distribution of the *B *obtained max*_z_*_∈_*_Z _LLR*(*z*) values under null hypothesis for circular clusters will be called the *scan empirical distribution*.

The statistical significance of the spatial scan statistic is done without pre-specifying the number of areas or the location of the most likely clusters, while adjusting for the multiple testing inherent in such a procedure. However, this adjustment is not done in an even manner for all possible cluster sizes, as will be shown later. The usual inference process compares the most likely cluster of observed cases with the all the circular most likely clusters of every possible size centered on every area of the study region. In this work it is presented a modification to the usual inference test of the spatial scan statistic: the observed most likely cluster found, with *k *areas, will be compared with only those most likely clusters of size *k *found in the randomized maps under the null hypothesis.

### Gumbel approximations

Through extensive numerical tests it was shown [9] that, under null hypothesis, the scan empirical distribution for circular clusters is approximated by the well-known Gumbel distribution

with parameters *μ *(mode) and *θ *(scale). Using this semi-parametric approach, the spatial scan distribution may be estimated using a much smaller number of Monte Carlo replications. For example, computing the max_*z*∈_*_Z _LLR*(*z*) values under null hypothesis for only 100 random maps, and obtaining their average and variance to calculate the mode and scale parameters, a semi-parametric Gumbel distribution is obtained, as accurate as a purely null empirical distribution produced after *B *= 10000 random Monte Carlo replications [9].

## Methods

One could be concerned with the fact that it should be more appropriate to compare the original MLC only with those MLCs of null hypothesis replicated maps that resemble as much as possible the original cluster, in terms of size, population and geographic location. An extreme instance of this situation would require that the comparison clusters are picked only among those (rarely occurring) random replications for which the MLCs are exactly the same as the original MLC. However, this task is computationally unfeasible, because an enormous number of replications is needed in order to select a sizable number of random simulations for which the simulated MLCs coincide with the original MLC. Therefore, those requirements must be somewhat relaxed. Requesting that the population is the same (regardless of other factors) may also be difficult, especially for maps with highly heterogeneous populations. A possibility then is to allow different location centers and populations, but requesting that the number of areas in the cluster is the same.

### Empirical distributions

The spatial scan statistic was designed to adjust for the multiple testing when evaluating clusters of different sizes and locations. This adjustment implicitly supposes that the scan distribution doesn't change when restricted for any given fixed cluster size. As will be shown, this assumption may not be true. Define scan*_k _*as the empirical distribution obtained from the scan empirical distribution which considers only clusters of size *k*. It is also show that the Gumbel semi-parametric approach could be extended to the scan*_k _*distributions.

### Frequency of cluster sizes

This subsection begins with two examples of maps with real data populations. The first map consists of 34 municipalities in the neighborhood of Belo Horizonte city in Brazil, with 6, 262 homicides cases during the 1998-2002 period, for a total population of 4, 357, 940 in 2000. The second map consists of 245 counties in 10 states and the District of Columbia, in the Northeastern U.S., with 58,943 age-adjusted deaths in the period from 1988 to 1992, for a population at risk of 29,535,210 women in 1990 [5].

**Figure 1:**
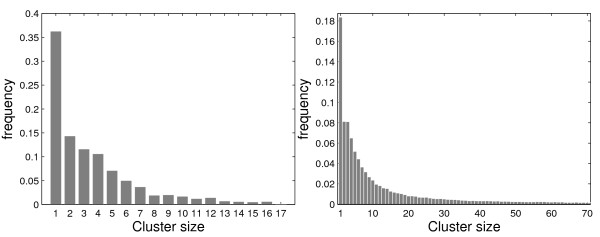
**Frequency distribution of the sizes of the most likely clusters found under *H*_0_, with 1,000, 000 Monte Carlo replications, for the Belo Horizonte metropolitan area (left) and the Northeastern US map (right)**.

For each map, 1,000,000 Monte Carlo replications under null hypothesis were conducted and the most likely clusters were found for each replication. The MLCs were classified according to their sizes and the frequencies of occurrence were displayed in the histograms of Figure 1. In both case studies, representing typical examples of aggregated area maps, the frequency of cluster sizes varies widely; clusters of very small size are much more common. This means that the shape of the scan empirical distribution depends mostly on the smallest cluster size. Consequently, the decision process about the significance of the original MLC of observed cases relies mainly on the behavior of very small clusters, regardless of its size. One could argue that this feature, in itself, should not represent a problem if it could be guaranteed that the scan*_k _*distributions were nearly identical for every value of *k*. However, as is shown in the next subsection, there are significant differences between the various scan*_k _*distributions.

### The Gumbel adjusted scan*_k _*distributions

As was done before for the empirical scan distribution, the Gumbel approximation for the scan*_k _*distribution is defined as the Gumbel*_k _*distribution. It was verified experimentally that this adjustment was adequate, for all cluster sizes in several different maps.

**Figure 2:**
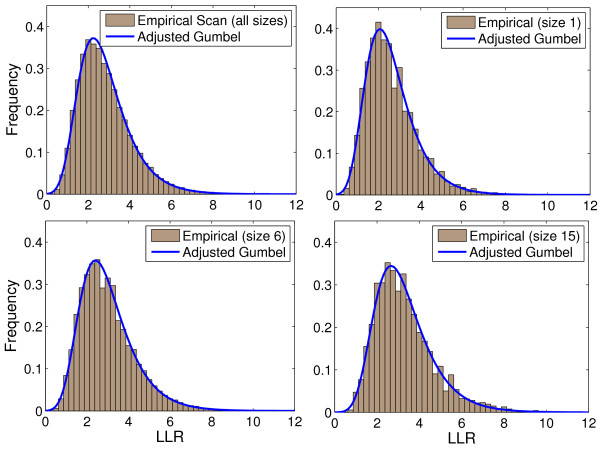
**The scan_*k *_distributions obtained from 1, 000, 000 Monte Carlo simulations in the Belo Horizonte etropolitan area map, and their respective Gumbel_*k *_adjusted distributions, for *k *= 1, 6 and 15**.

For the Belo Horizonte map, Figure 2 shows the scan*_k _*distributions taken from 1, 000, 000 Monte Carlo simulations and their respective Gumbel*_k _*adjusted distributions, for *k *values 1, 6 and 15.

**Figure 3:**
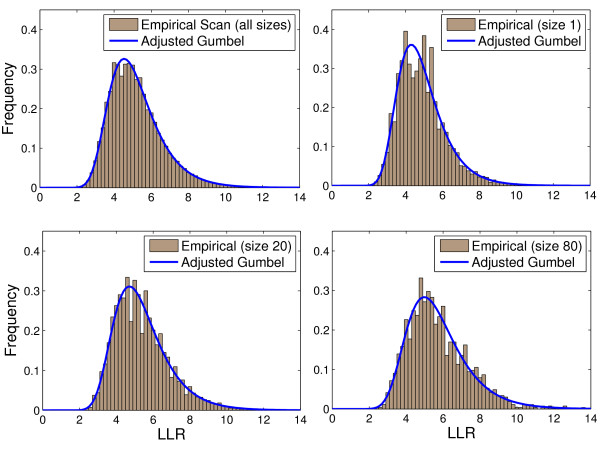
**The scan_*k *_distributions taken from 1, 000, 000 Monte Carlo simulations in the Northeastern US map, and heir respective Gumbel_*k *_adjusted distributions, for *k *= 1, 20 and 80**.

Similarly, the same procedure was performed considering the map of the Northeastern US, for *k *values 1, 20 and 80 as seen in Figure 3.

**Figure 4:**
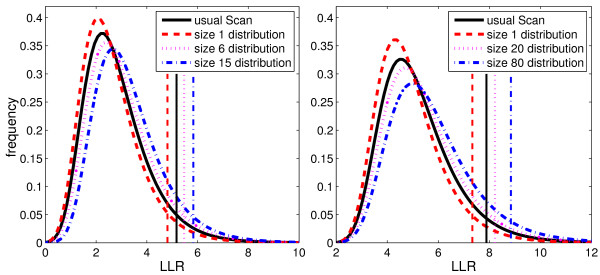
**The Gumbel adjusted distribution and the Gumbel_*k *_distributions for several values of *k*, with their respective *α *= 0.05 critical values, for the Belo Horizonte metropolitan area (left) and the Northeastern US (right)**.

From the results of the 1, 000, 000 Monte Carlo simulations for the map of Belo Horizonte metropolitan area, it was observed that the critical values for the Gumbel*_k _*distributions increase monotonically as the index *k *increases from 1 to the maximum value 17. The Gumbel adjusted distribution and the Gumbel*_k _*distributions for *k *= 1, 6 and 15 are displayed in the same graph on the left of Figure 4. The *α *= 0.05 critical values for the four corresponding distributions are also shown.

For the Northeastern US map, similar results were observed when the Gumbel adjusted distribution and the Gumbel*_k _*distributions for *k *= 1, 20 and 80 were put together in the graph on the right of Figure 4, with the corresponding *α *= 0.05 critical values.

Those two examples show that the Gumbel*_k _*distributions change steadily with the size *k*, and are significantly different from the Gumbel adjusted distribution including all cluster sizes.

### Data-Driven Inference

In its original formulation, the circular scan calculates the significance of the most likely cluster based on the following question: "Given that the candidate cluster found has *LLR *= *x*, what is the probability of finding a most likely cluster under the null hypothesis with *LLR *>*x *?"

As will be seen in this section, clusters of small size constitute the majority among the MLCs found, and have the highest influence in the determination of the scan empirical distribution. In the situation when the MLC has large size, its inference will be made basically by the behavior of small clusters.

In this section it is proposed to use the information about the size of the observed MLC in the inference test. In this case, the following question is formulated: "Given that the candidate most likely cluster has *LLR *= *x *and contains *k *areas, what is the probability to find, under the null hypothesis, a cluster formed by *k *regions with *LLR > x*?"

The proposal of this paper takes into account the cluster size, as follows: given that the circular scan has found the observed MLC with *k *regions, then its statistical significance is still obtained through Monte Carlo simulations, but selecting only those replications for which the MLC solutions have exactly *k *areas. The empirical scan distribution that considers MLC solutions of any size is replaced by the scan*_k _*empirical distribution with MLCs of size exactly equal to *k*.

However, when the p-value is much smaller or much larger than the significance level *α*, there is no change in the decision to reject, or not, the null hypothesis. Thus, only the more difficult decision process is of interest, namely when the computed p-value is close to the significance level *α*.

## Results

In this section, numerical experiments are made showing that the proportions of rejections of the null hypothesis differ noticeably, by employing the usual critical value, compared with using the data-driven critical values. It is also shown that the computational cost of estimating the data-driven critical value may be reduced through the use of a simple interpolation.

### Critical values variability

To assess the variability in the estimation of the critical values of each scan*_k _*distribution, another batch of Monte Carlo simulations was conducted. First a set *S*_0 _of 1, 000, 000 random replications under null hypothesis was computed and the usual critical value *v*_0 _was determined for the *α *= 0.05 significance level employing the empirical scan distribution. Next, for every value of size *k *the empirical scan*_k _*distribution and the corresponding critical value *v_k _*at the *α *= 0.05 significance level was determined.

Following, 100 sets *S_i_*, *i *= 1, ..., 100 of 1, 000, 000 random replications each were computed under null hypothesis. For each set *S_i _*and for every value of size *k *the corresponding empirical scan*_k _*distribution for the set *S_i _*was computed, and called scan*_ki_*. The proportion *p_ki _*(respectively *q_ki_*) of computed MLCs' LLR values larger than *v*_0 _(respectively *v_k_*) in the distribution scan*_ki _*was obtained, for every value *k *and *i *= 1, ..., 100. For every value of *k*, the 100 values *p_ki _*(respectively *q_ki_*), for *i *= 1, ..., 100, were used to build the 95% confidence intervals *U_k _*(respectively *D_k_*), thus estimating the error bar in the proportion of rejection of the null hypothesis through the usual (respectively data-driven) inference process.

**Figure 5:**
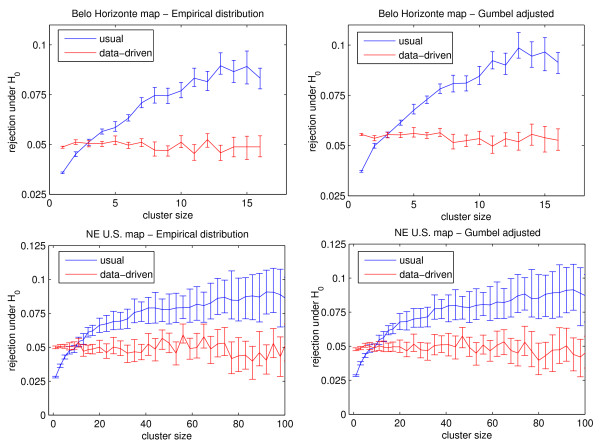
**The proportion of null hypothesis rejection, for the empirical (left) and Gumbel adjusted (right) distributions, for the Belo Horizonte metropolitan area (above) and the Northeastern US map (below)**. The 95% non parametric confidence intervals are built from 100 Monte Carlo experiments of 1,000,000 replications each, using the usual (blue) and the data-driven (red) inference processes.

The graphs on the left part of Figure 5 show the results using the data for the Belo Horizonte metropolitan area (above) and the US Northeastern map (below). The graphs in blue color show the error bars for the 95% usual non parametric confidence intervals *U_k_*, for every size *k*, showing the average and the variability of the proportion of likelihood ratios higher than the usual critical value *v*_0_, which employs MLCs of every size *k *in the empirical scan distribution. Otherwise, the graphs in red color show the error bars for the 95% data-driven non-parametric confidence intervals *D_k_*, for every size *k*, showing the average and variability of the proportion of likelihood ratios higher than the data-driven critical values *v_k_*, which separates MLCs of different sizes *k *into their corresponding empirical scan*_k _*distributions. For each dataset, the blue and red error bars are clearly distinct, showing the differences between the usual and data-driven inferences. A similar procedure was used to compute the 95% non parametric confidence intervals for the corresponding adjusted Gumbel*_k _*distributions, instead of the empirical scan*_k _*distributions. The corresponding sets of error bars for the Gumbel adjusted distributions are displayed in the right part of Figure 5, for the Belo Horizonte metropolitan area (above) and the US Northeastern map (below). From Figure 5 it is noted that the values obtained through the data-driven inference are substantially closer to the 0.05 level, for both maps. The usual approach's rejection rate of about 0.03 to 0.04 for small clusters means it is not rejecting enough small clusters, thus returning too many false positives. The opposite happens for large clusters.

### Practical evaluation of critical values

From a practical viewpoint, one could be concerned with the large number of simulations necessary to obtain a reasonable number of replications for which their MLCs have exactly the size of the observed MLC, in order to estimate the data-driven critical value. It is shown that, through interpolation of the *v_k _*critical values for the values of size *k *close to the observed MLC size, a fairly small number of replications produce a consistent critical value estimate.

**Figure 6:**
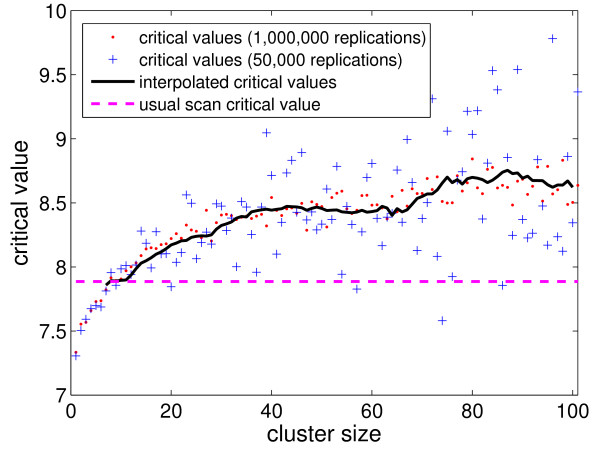
**Data-driven critical values for the Northeastern US map for the various *k *size values, using 1,000,000 (respectively 50,000) replications, displayed as red dots (respectively blue crosses)**. The solid black curve represents the moving average, of window size 20, of the critical values associated to each size *k *> 10 using the 50,000 replications set (blue crosses).

Figure 6 shows the data-driven critical values for the Northeastern US map for the *k *size values, using 1,000,000 (respectively 50,000) replications, displayed as red dots (respectively blue crosses). The solid black curve represents the moving average, of window size 20, of the critical values for each size *k *> 10 using the 50,000 replications set (blue crosses). As can be seen in Figure 6, the moving averages fall approximately within the 1,000,000 set's obtained critical values (red dots). The horizontal dashed line indicates the usual critical value. This very simple scheme is thus sufficiently stable, allowing the use of a small number of Monte Carlo replications to estimate the data-driven critical values. For smaller maps (as the Belo Horizonte metropolitan area map), the computational effort is lower, and the data-driven critical values are easier to calculate.

## Conclusions

The classical inferential process used in the inference of spatial clusters employing Kulldorff's Spatial Scan statistic considers all the most likely clusters found in Monte Carlo replications under the null hypothesis in order to build the empirical distribution of the likelihood ratio, regardless of cluster size and location. A potential disadvantage of this approach is the implicitly assumed independence of the log likelihood ratio distributions, when restricted to the various sizes of the most likely cluster found. It was shown, through numerical experiments, that this assumption is not true for commonly occurring real data maps. Given that the observed most likely cluster has size *k*_0_, that means that the classical inference process computes its significance based on the behavior of most likely clusters whose sizes are different from *k*_0_.

In this work an alternative was proposed, the data-driven inference, which takes into account only those most likely clusters found whose size is identical to the size of the observed most likely cluster. This approach employs a more specific comparison, thus avoiding that the behavior of clusters of very small size are used to decide if a large observed most likely cluster is considered significant, for example.

As the number of most likely clusters of certain specific size found in randomized maps is smaller than the total number of Monte Carlo replications, a concern may arise that the computational effort to compute the significance with the data-driven inference process should be very high. However, it was shown that using the Gumbel semi-parametric approach and simple interpolation techniques this effort could be mitigated. How many simulations are necessary to provide an acceptable confidence interval for the critical values for each value of size k? It would be very difficult to find a simple formula fitted for every kind of map. Given a particular map, it is suggested to apply the method described in the "Practical evaluation of critical values" section, running sequentially several sets of simulations with an increasing number of Monte Carlo replications, and waiting for the interpolated critical values curve to numerically stabilize.

For each value of size *k *there are different distributions of empirical values; in all the studied examples, the overall shape of those distributions varies smoothly as *k *varies, and it even seems reasonable to conjecture that their averages increase monotonically as the value of *k *increases. However it is plausible to imagine an hypothetical situation where those changes become abrupt with the variation of *k*, characterizing instability of the null hypothesis. This instability should impact even more the usual inference, because the relative importance of the different values of k would not be identified, as was done in the present analysis. This kind of situation has not yet been experienced, although, and the experiments of the previous section may serve to assess that kind of instability in a particular map.

It should be stressed that the present paper's strategy of employing the size (number of areas inside the cluster) as the criterion to compare the most likely clusters is still subject to bias due to the heterogeneous population distribution. A solution for this problem should be the use of both variables, size and population, which would be very expensive, as discussed before.

The proposed method is more useful when the computed p-value using the classical inference is close to the *α *significance level, otherwise there will be no change in the decision process. In this situation, it is recommended that the data-driven inference should be performed, especially when the observed most likely cluster has relatively large size.

Numerical experiments were also performed using the data-driven inference for space-time cluster detection, considering not only the number of areas inside the cluster, but also the length of the time interval of the cylinders [10]. Preliminary results suggest that the differences in the critical values are even more pronounced than in the purely spatial setting.

The data-driven inference could be applied to case/controls point data set clusters, taking into account the number of cases and the population inside the clusters. There are three options to consider for the data-driven inference in this type of dataset, namely those based on the number of cases, population, or both. When based on the number of cases, provided that the number of cases is small, the data-driven inference follows the same lines of the present paper. Otherwise, when the number of cases is large or when the data-driven inference is based on the population, some kind of interpolation scheme should be used.

Another extension should consider irregularly shaped clusters [11] instead of circular clusters. These ideas will be discussed in a future work.

## Competing interests

The authors declare that they have no competing interests.

## Authors' contributions

All the authors contributed to the methodology used in the study, wrote the necessary computer programs, conducted the simulations and data analysis, and drafted the manuscript. All authors have read and approved the final manuscript.
